# Genome alteration of *Leishmania orientalis* under Amphotericin B inhibiting conditions

**DOI:** 10.1371/journal.pntd.0012716

**Published:** 2024-12-17

**Authors:** Pornchai Anuntasomboon, Suradej Siripattanapipong, Sasimanas Unajak, Kiattawee Choowongkomon, Richard Burchmore, Saovanee Leelayoova, Mathirut Mungthin, Teerasak E-kobon

**Affiliations:** 1 Department of Genetics, Faculty of Science, Kasetsart University, Bangkok, Thailand; 2 Omics Center for Agriculture, Bioresources, Food, and Health, Kasetsart University (OmiKU), Bangkok, Thailand; 3 Department of Microbiology, Faculty of Science, Mahidol University, Bangkok, Thailand; 4 Department of Biochemistry, Faculty of Science, Kasetsart University, Bangkok, Thailand; 5 Glasgow Polyomics, College of Medical, Veterinary and Life Sciences, University of Glasgow, Glasgow, United Kingdom; 6 Department of Parasitology, Phramongkutklao College of Medicine, Bangkok, Thailand; U.S. Food and Drug Administration and Center for Biologics Evaluation and Research, UNITED STATES OF AMERICA

## Abstract

Amphotericin B (AmB) is a potent antifungal and antiparasitic medication that exerts its action by disrupting the cell membrane of the leishmanial parasite, leading to its death. Understanding the genetic alterations induced by Amphotericin B is crucial for gaining insights into drug resistance mechanisms and developing more effective treatments against *Leishmania* infections. As a new *Leishmania* species, the molecular response of *Leishmania orientalis* to anti-leishmanial drugs has not been fully explored. In this study, *Leishmania orientalis* strain PCM2 culture was subjected to AmB exposure at a concentration of 0.03 uM over 72 hours compared to the control. The genomic alteration and transcriptomic changes were investigated by utilising the whole genome and RNA sequencing methods, followed by the analysis of single nucleotide polymorphisms (SNPs), differential gene expression, and chromosomal copy number variations (CNVs) assessed using read depth coverage (RDC) values across the entire genome. The chromosomal CNV analysis showed no significant difference between *L*. *orientalis* from the control and AmB-treated groups. The distribution of SNPs displayed notable variability, with higher SNP incidence in the control group compared to the AmB-treated group. Gene ontology analysis unveiled functions of the SNPs -associated genes involved in transporter function, genetic precursor synthesis, and purine nucleotide metabolism. Notably, the impact of AmB treatment on the *L*. *orientalis* gene expression profiles exhibited diverse expressional alterations, particularly the downregulation of pivotal genes such as the tubulin alpha chain gene. The intricate interplay between SNPs and gene expression alterations might underscore the complex regulatory networks underlying the AmB resistance of *L*. *orientalis* strain PCM2.

## Author summary

Leishmaniasis is a neglected tropical disease caused by parasites of the Leishmania genus, and treatments often rely on drugs like Amphotericin B (AmB). This study focuses on Leishmania orientalis, a newly recognized species, to better understand its molecular responses to AmB, which could shed light on drug resistance mechanisms. Using advanced genomic and transcriptomic techniques, the authors analyzed how L. orientalis responds to AmB treatment over 72 hours. Interestingly, while there were no significant changes in chromosomal structure, the authors observed differences in single nucleotide polymorphisms (SNPs) and gene expression patterns. The control group showed more genetic variability than the treated group, and several key genes involved in critical cellular functions were downregulated after treatment. These findings suggest that L. orientalis might regulate its genetic and molecular machinery in complex ways to survive drug exposure. By unraveling these mechanisms, this research contributes to understanding Leishmania biology and supports the development of more effective treatments for this neglected disease.

## 1. Introduction

The protozoan parasite *Leishmania* is globally distributed and responsible for many disorders, including cutaneous, mucocutaneous, and visceral leishmaniasis. Vaccines against *Leishmania* infections are not currently available [1]. The genomes of several *Leishmania* species are complex, varying in size, number of chromosomes, and number of protein-coding genes [2,3]. The genes are also arranged uniquely, many transcribed as polycistronic transcripts [4–6]. In addition, the *Leishmania major* genomes contain repetitive DNA sequences lower than 10%, while genomic analysis of *Trypanosoma cruzi* reveals a substantial presence of repetitive DNA sequences, comprising a noteworthy 51.25% of its entire genome [7]. A previous report regards the leishmanial parasite as a "mainly diploid" or aneuploid organism [8]. Individual cells may exhibit two or more ploidy states within the *Leishmania* cell population, i.e., monosomic, disomic, or trisomic, resulting in highly variable chromosomal contents between cells. Mosaic aneuploidy was reported to be an inherent characteristic of this parasite [9]. The chromosomal alteration was also found under drug pressure, such as trivalent antimony (SbIII) resistance in *L*. *braziliensis* (MHOM/BR75/M2904) and *L*. *panamensis* (MHOM/COL/81L13*)*, which revealed discrepancies in somy, copy number variations of specific antimony-related genes, also deletions and duplications in chromosomes without somy changes. Notably, a direct correlation between chromosomal and local copy number variation and gene expression was primarily observed in *L*. *braziliensis* [10], various mechanisms through which the gene copy numbers related to methotrexate resistance were altered, including gene deletion and formation of extrachromosomal circular or linear amplicons in *L*. *major* [11]. Supernumerary chromosomes 6 and 11 were observed in Nelfinavir-resistant *L*. *donovani* amastigotes, resulting in an increased expression of ABC transporters. Furthermore, high-resolution electron microscopy and intracellular polyphosphate measurements indicated a higher abundance of cytoplasmic vesicular compartments, known as acidocalcisomes, in these resistant parasites [12].

Amphotericin B is a versatile medication with antifungal and antiparasitic properties, rendering it effective against various parasites, including *Leishmania* [13]. This polyene drug operates by attaching to the parasite’s cell membrane, prompting the creation of pores and leading to the leakage of crucial cellular components [14]. The leishmaniasis treatment with Amphotericin B is highly regarded as a potent therapeutic option, especially when conventional treatments like pentavalent antimonials have proven ineffective or are unsuitable due to contraindications [15–17]. Nonetheless, it has the potential to induce severe side effects, including (causing damage to the kidneys) and infusion-related reactions [18,19]. Several studies examined the effects of Amphotericin B on *Leishmania* using different approaches [14,19–24]. Global RNA sequencing (RNA-Seq) has proven to be a potent and versatile technique for elucidating comprehensive changes in the transcriptome of *Leishmania* parasites under various conditions, including exposure to drug pressure. This high-throughput approach enables a systematic and in-depth analysis of the transcriptional landscape, providing a deeper understanding of the molecular mechanisms underlying the parasite dynamic responses and adaptations in the face of AmB-induced stress [25].

A previous review article considered leishmaniasis an imported disease in Thailand before 1999 [26]. Since then, autochthonous leishmaniasis has been reported in both immunocompetent and immunocompromised patients, especially those with HIV/AIDS [26]. The new *Leishmania* species, *Leishmania orientalis* (formerly *Leishmania siamensis*), consisting of two lineages, were identified in Thailand. The parasites cause both cutaneous leishmaniasis (CL) and visceral leishmaniasis (VL) in Thai patients [27–29]. A preliminary genome study of *Leishmania* spp. in Thailand revealed that the genetic diversity of these species varied considerably, even among the same species found in different geographical regions of Thailand [30]. However, the response to drugs in *L*. *orientalis* in Thailand remains limited. This limited drug response presents significant challenges for treating and controlling the parasite’s spread in the region. Consequently, further research is crucial to improve understanding of drug resistance mechanisms and develop more effective treatment strategies. This study aimed to identify an initial stage of molecular adaptation of *L*. *orientalis* strain PCM2 after culturing in the Amphotericin B-treated and Amphotericin B-free media using the whole genome and transcriptome sequencing and bioinformatics analysis. Understanding the alterations in the genomic and transcriptomic changes of *L*. *orientalis* under the Amphotericin B pressure could offer valuable insights into potential drug resistance mechanisms and contribute to the development of more targeted and effective therapeutic strategies for combating leishmaniasis.

## 2. Materials and Methods

### 2.1. Amphotericin B susceptibility test of Leishmania orientalis

*L*. *orientalis* strain PCM2 at the promastigote stage was grown at 26°C in Schneider’s insect medium (MilliporeSigma, US) at pH 6.7 with a 20% final concentration of heat-inactivated fetal bovine serum (HIFBS, Gibco, US). To determine the half-maximal inhibitory concentration (IC50), Amphotericin B (Thermo Fisher Scientific, US) was treated at the concentrations of 0.01, 0.05, 0.1, 1, 5, 10, 50 and 100 μM with *L*. *orientalis* strain PCM2 (10^5^ cells) in the 96-well microplates and incubated at 26°C for 72 hours. After adding alamarBlue (Thermo Fisher Scientific, US) for 24 hours, the absorbance was measured using the E.Z. Read 400 (Microplate reader; Biochrom, US), and IC50 values were determined. The IC50 values were determined by plotting the concentration-response data on a dose-response, typically a sigmoidal curve. The concentration at which the response was reduced by 50% was identified to calculate the IC50 value. Non-linear regression analysis using the four-parameter logistic equation in the Prism GraphPad program was used to fit the data and estimate the IC50 value along with its associated confidence intervals.

### 2.2 *L*. *orientalis* culturing and Amphotericin B treatment

The *L*. *orientalis* strain PCM2 promastigotes were cultured in triplicates in Schneider’s insect medium supplemented with 20% Fetal Bovine Serum (FBS) (Thermo Fisher Scientific, US) at pH 6.7. The culture flask was maintained at 26°C in an incubator for 72 hours until the parasites reached the procyclic promastigote stage. Subsequently, a medium containing Amphotericin B was prepared, achieving a final concentration of 0.03 μM (10X diluted from the IC50 value), and added to a new culture flask containing 60 ml of fresh medium. The cells were transferred by gently swirling the original flask to ensure even distribution, followed by adding approximately 2.5 ml of the cells into the new culture flask. The culture was then further incubated at 26°C for another 72 hours. The culture was centrifuged at 1,000 xg for 10 minutes to harvest the parasite pellets and discard the supernatant. The pellet was washed twice with PBS (Phosphate Buffered Saline) (Thermo Fisher Scientific, US) and then stored at -20°C for further use.

### 2.3 Genomic DNA preparation and whole genome sequencing

Genomic DNAs were prepared from the promastigotes at the late logarithmic phase. The promastigote pellets were washed with ultrapure water and resuspended in 1 ml of lysis buffer (10 mM Tris, 10 mM KCl, 10 mM MgCl_2_, 0.5 M NaCl, 2 mM EDTA, and 0.5% SDS) and 20 ul of proteinase K solution (20 mg/ml) (Vivantis technologies, Malaysia). The samples were incubated at 56°C for 30 minutes, and the same volume of chloroform: isoamyl alcohol (24:1) was added to the sample before vigorous shaking for 10 minutes. The samples were centrifuged at 10,000 rpm at room temperature for 10 minutes, and the upper aqueous phase was collected. 10 μl of the RNAse solution (20 mg/ml) (Vivantis technologies, Malaysia) was added to the samples, and the reaction was incubated at room temperature for 3 minutes. After the RNase treatment, one time of chloroform: isoamyl alcohol (24:1) was added again, and the samples were shaken vigorously for 10 minutes. The samples were centrifuged at 10,000 rpm at room temperature for 10 minutes, and the upper aqueous phase was collected. The genomic DNAs were precipitated overnight in 200 μl of 4 M ammonium acetate and 800 μl absolute ethanol at -70°C. The extracted DNA fractions were centrifuged at 10,000 rpm at 4°C for 10 minutes before washing twice with 70% ethanol. DNA samples were air-dried at room temperature for 30 minutes and resuspended in TE buffer (10 mM Tris-HCl pH 8.0 and 0.1 mM EDTA). DNA samples were qualified by measuring absorbance at 260/280 nm and quantified by measuring absorbance at 260 nm using NanoDrop One/OneC Microvolume UV-Vis Spectrophotometer (Thermo Fisher Scientific, US) and separation on 1% agarose gel electrophoresis. The samples were kept at −70°C until use. The genomic DNAs were used to construct 150-bp genomic DNA libraries, and the paired-end genome sequencing was performed using the Illumina HiSeq 2000 platform (Illumina, US). Raw sequence reads were quality checked, filtered, and trimmed by using the fastp program version 0.23.2 [31] with the quality filter of 30, and the reads were mapped to the *L*. *orientalis* strain LSCM4 genome (GenBank accession number GCA_017916335.1) using BWA-MEM version 0.7.17 [32].

### 2.4 Total RNA preparation and RNA sequencing

All three biological replicate samples (the AmB-treated and AmB-free conditions) were collected as described in the previous section. Following a rigorous protocol, total RNA was extracted from the biological samples using the RNeasy Plus Mini Kit (Qiagen, Germany). The protocol involved lysing and homogenising the sample, passing the lysate through a gDNA Eliminator spin column, adding ethanol to the flow-through, and applying the sample to an RNeasy MinElute spin column. The purified RNA was ultimately eluted in RNase-free water. RNA samples were subjected to the preparation of the Illumina DNA sequence libraries following the manufacturer’s specifications, including RNA fragmentation, first-strand cDNA synthesis via reverse transcription, generation of double-stranded cDNA, end repair, and subsequent ligation with Illumina sequencing adapters. Size selection of ligated cDNA fragments was achieved through gel electrophoresis or bead-based purification, and library enrichment via polymerase chain reaction (PCR) introduced sample-specific index sequences for multiplexing and amplifying library materials for sequencing. Quality control measured and ensured the validation of fragment size distribution, using gel electrophoresis to confirm that cDNA fragments were within the optimal size range for sequencing. Accurate library quantification was performed using a fluorometric method to ensure that the library concentration was optimal for sequencing efficiency and data quality.

### 2.5 Aneuploidy analysis of the *L*. *orientalis* genomic reads

The analysis of aneuploidy in the *Leishmania* genomes was carried out following the trypanosomatids methods and protocols guideline [33]. This comprehensive analysis of aneuploidy employed the read depth coverage (RDC) determination for each genomic position of the *L*. *orientalis* genomes from the AmB-treated and AmB-free conditions. RDC was calculated by dividing the total number of sequencing reads that align to a specific position in the genome by the size of the genomic region or the length of the reference sequence at that position. The BWA-MEM program version 0.7.17 [32] performed mapping of the preprocessed reads to the reference *L*. *orientalis* genome, resulting in the generation of an alignment file in Sequence Alignment/Map (SAM) format and converted to the Binary Alignment Map (BAM) format using the SAMtools program version 1.11 [34]. The read depth coverage for each genomic position in individual chromosomes was determined through the bedtools program version 2.30.0 [35,36]. The genome coverage and chromosomal somy were estimated based on the mean genome coverage calculated by summing the read depth coverage for each position in the genome and then dividing by the total number of positions in the genome. The calculation resulted in the mean RDC for the entire genome. The chromosomal somy table was generated to display the normalised copy numbers for each chromosome. This normalisation was based on the ratio between each chromosome’s mean genome RDC and the mean RDC. For improved accuracy in the somy estimation, the analysis was restricted to the coding sequence (CDS) region, excluding non-CDS repetitive regions and "N"-rich blocks in the reference genome. To estimate chromosomal somy based on allele frequency analysis, an index and sequence dictionary were generated to facilitate Genome Analysis Toolkit (GATK) processing version 4.4.0.0 [37], including marking PCR duplicated reads. Subsequently, the VCF file containing all SNP positions was generated using the GATK program [37]. Only the heterozygous positions were selected from this step, and allele frequency ratios were calculated for the entire genome and each individual chromosome. For visualisation and analysis of the chromosome copy number variation (CCNV), the R programming scripts [38] were employed, and the ggplot2 package [39,40] was utilised to generate informative and insightful plots.

### 2.6 Genomic variant identification and analysis

In order to discern single nucleotide polymorphisms (SNPs) between genomes of the *L*. *orientalis* samples cultivated under the AmB-treated and AmB-free conditions, the GATK4 version 4.4.0.0 [37] was used, primarily employing the haplotype caller module for SNP identification. Subsequently, the raw variant calls underwent stringent filtering, adhering to recommended parameter thresholds (Q.D. < 2.0, QUAL < 30.0, SOR > 3.0, F.S. > 60.0, M.Q. < 40.0, MQRankSum < -12.5, and ReadPosRankSum < -8.0). Variants were selectively filtered within each experimental condition for a focused analysis of the SNP impacts within the coding regions of the *L*. *orientalis* genomes. Following this, the variant calls from both conditions were integrated, generating a comprehensive variant dataset. The merged variant dataset served as the input for the SnpEff program version 5.1 [38], facilitating the annotation and prediction of the potential functional effects of the identified genetic variants on specific genes within the *L*. *orientalis* genomes.

### 2.7 Differential expression analysis of the transcriptome data

The raw RNAseq data was acquired in the fastq format following the RNA sequencing process. Subsequently, the quality validation and adapter content trimming were carried out using the Fastp program version 0.23.2 [31], which also addressed the removal of over-represented sequences. Transcript abundance was quantified using the Kallisto program version 0.46.1, an efficient pseudo-alignment-based tool [41]. Kallisto employed the *L*. *orientalis* strain LSCM4 genome (GenBank accession number GCA_017916335.1) as a reference for quantification. Pseudoalignment of sequencing reads to the transcriptome was performed using Kallisto with default parameters. This step generated transcript-level abundance estimates in transcripts per million (TPM) for each sample. Before differential expression analysis, transcript-level abundance estimates were normalised, and count data were modelled using the Wald test to assess the significance of expression differences between conditions. The differential expression analysis was conducted to identify the genes influenced by the Amphotericin B treatment against *L*. *orientalis* strain PCM2 by applying the Wald test with a stringent q-value threshold of < 0.05, representing the minimum false discovery rate (FDR). The principal component analysis (PCA) and hierarchical clustering heatmap were performed by the Sleuth package [42] in R [43]. For the RNA variant calling method, STAR (Spliced Transcripts Alignment to a Reference) aligner version 2.7.11a [44] and GATK4 program version 4.4.0.0 [37] were used for the comprehensive identification of genetic variants from RNA-seq data. Initially, high-quality RNA-seq reads from the experimental samples were aligned to the reference genome using STAR, which incorporated an accurate splice junction mapping algorithm. Following alignment, the resulting BAM files were processed through GATK4. Preprocessing steps included base recalibration, indel realignment, and duplicate removal. Subsequently, variant calling was performed using GATK4’s HaplotypeCaller tool, which employed a local *de novo* assembly approach for detecting single nucleotide polymorphisms (SNPs) and insertion-deletion (indel) variants. The identified variants were then filtered based on quality metrics, allele depth, and mapping quality to ensure the reliability of the final variant calls.

### 2.8 Gene annotation and gene ontology enrichment analysis

The *L*. *orientalis* strain PCM2 genome was comprehensively annotated using the COMPANION web server (https://companion.sanger.ac.uk) [45]. This annotation process involved referencing the *L*. *orientalis* strain LSCM4 genome (MHOM TH 2014) as a template, with default parameters, including a minimum required match length of 500 bp and a minimum required match similarity of 85%. Additionally, to augment the annotation of hypothetical genes and enhance the identification of uncharacterised proteins, the SWISS-MODEL program (https://swissmodel.expasy.org/) [46] predicted the protein structures by incorporating structural templates available from the AlphaFold Protein Structure Database [47]. The AlphaFold Protein Structure Database is an extensive database of high-accuracy protein-structure predictions developed by DeepMind, and EMBL-EBI has provided open access to over 200 million predicted protein structures from the human proteome and 47 organisms, including *Leishmania* species [47,48]. This approach focused on identifying proteins with substantial structural similarity and meeting specific criteria, including the Global Model Quality Estimation (GMQE) score exceeding 0.80 and coverage greater than 80%.

To further elucidate the functional annotations of the identified genes, the PANNZER2 server [49] was deployed to assign the gene ontology or GO categories. The enriched term hierarchical clustering employed Jaccard’s similarity index to assess the similarity and diversity of sample sets. The index was computed as the ratio of the intersection size of two sets to their union size. The hierarchical clustering was also performed using the Ward algorithm to minimise variance within each cluster. The data obtained from PANNZER2 were subsequently analysed and visualised using R packages, including 1) AnnotationHub [50] to access genomic data resources from various databases, which included the genome-wide annotation for Malaria (org.Pf.plasmo.db) via the Bioconductor package version 3.8, 2) ClusterProfiler tools for functional enrichment analysis (i.e., gene set enrichment analysis (GSEA), over-representation analysis (ORA), and pathway analysis) [51], 3) GOSemSim [52] for the semantic similarity measurement of the GO terms and included functions for calculating the semantic similarity between GO terms based on their annotations, and 4) enrichplot [53] provided visualisation tools for functional enrichment analysis results and included functions for creating bar plots, dot plots, and heatmaps to visualise enriched gene sets.

### 2.9 K-mer analysis for transcriptomic responses to the Amphotericin B treatment

The k-mer analysis workflow was employed to unravel the transcriptomic shifts induced by the Amphotericin B (AmB) treatment in *L*. *orientalis* strain PCM2. High-quality RNA-seq data were obtained after stringent quality control with the fastp program. Jellyfish was used to generate k-mers of length 31 from both control and AmB-treated samples. K-mers with counts below six times were filtered out to enhance the signal-to-noise ratio. To visualise and compare the k-mer distribution between the two conditions, pandas and matplotlib libraries in Python were employed to facilitate quantitative analysis and visualisation. The unique k-mers exclusive to each experimental condition were identified and collected. Using the Trinity assembler, these were then used as seeds for de novo assembly. BLASTn (Nucleotide BLAST) [54] with a local RNAseq dataset (https://ftp.ncbi.nlm.nih.gov/blast/db/refseq_rna.00-13) was employed to annotate the functionality of these RNA contigs. Finally, the SankeyMATIC [55] plot was generated to visualise the annotated genes.

### 2.10 Targeting *Leishmania* RNA viruses 1 and 2 in *L*. *orientalis* strain PCM2

The multi-pronged approach was used to identify *Leishmania* RNA viruses (LRVs) in the genome of *L*. *orientalis* PCM2. The LRV 1 and 2 (GenBank: OQ673100.1 and U32108.1) from the National Center for Biotechnology Information (NCBI) database were used as reference sequences. The RNAseq reads were aligned against the LRV references using Burrows-Wheeler Aligner (BWA) version 0.7.17. For confirmation, *de novo* assembly using Trinity assembler version 2.9.1 was employed to construct the LRV RNA contigs. LRV references were used to create the local LRVs database and compared with the assembled LRV contigs using the local BLASTn tools.

## 3. Results

### 3.1 Genome alteration of *L*. *orientalis* strain PCM2 treated with Amphotericin B

**Fig 1:**
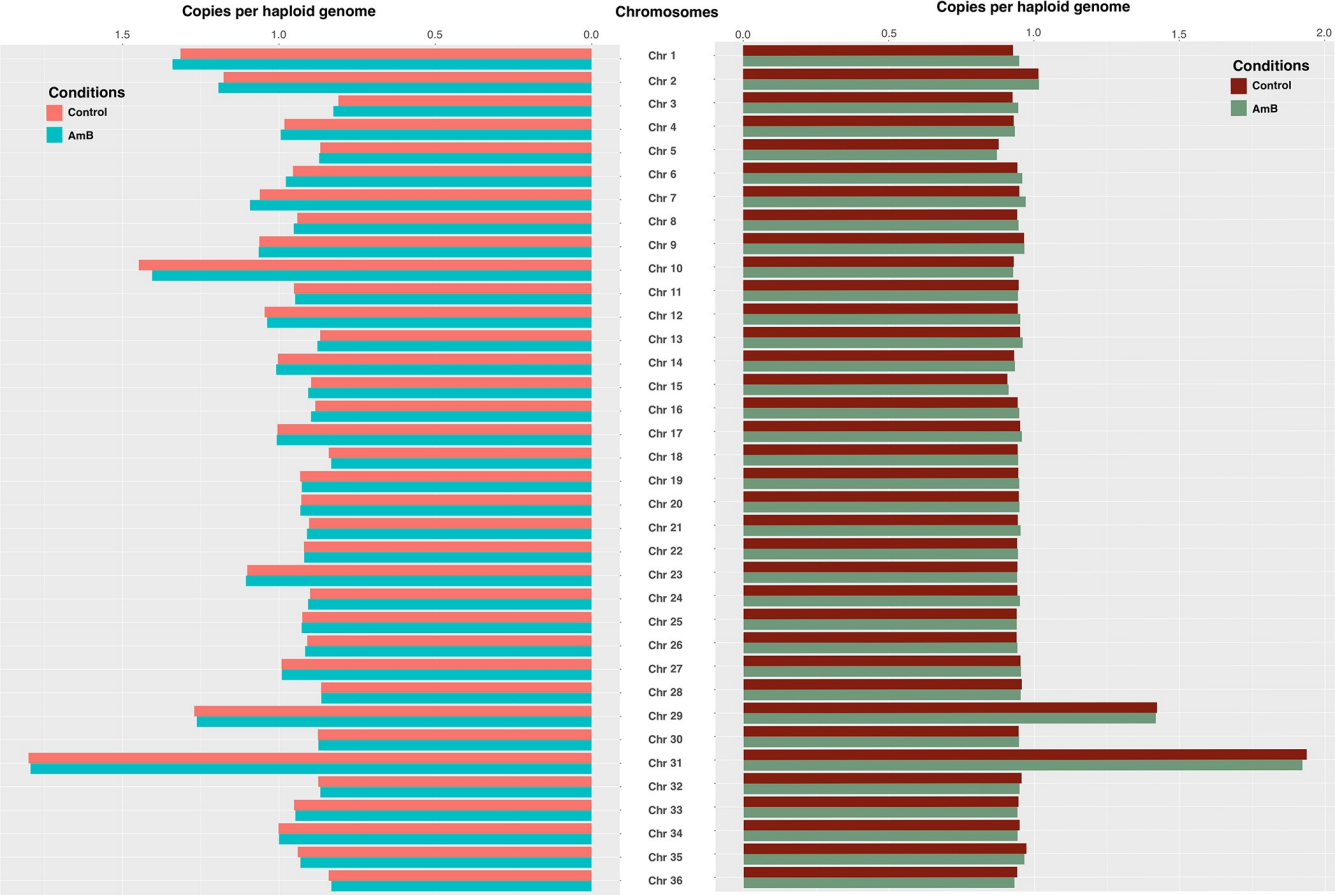
The graphs represented the estimation of the chromosome number and number of chromosomal copies of *Leishmania orientalis* strain PCM2 from the genomic reads based on the read depth coverage (RDC) per haploid genome compared between the *L*. *orientalis* growth under the 0.03 μm AmB-treated (AmB) and AmB-free (control) conditions in triplicates. The RDC values were calculated from the whole genome in triplicates (left panel) and from only the coding sequences (right panel). The x-axis showed the number of chromosomal copies per haploid genome, and the y-axis showed the *L*. *orientalis* chromosomes.

The IC50 test was conducted on *L*. *orientalis* PCM2 treated with 0.03 μM Amphotericin B (AmB) (**S1 Fig**), a medication frequently used for visceral leishmaniasis. Different *Leishmania* species and isolates had various responses to the AmB treatment [20,23,56,57]. Read depth coverage (RDC) and mean genome coverage were used to study chromosome aneuploidy in the *Leishmania* genomic analysis. Higher RDC indicates that a specific genomic region or chromosome has been sequenced more thoroughly, while lower RDC suggests less coverage or sequencing depth for that region. The triplicate treatment of *L*. *orientalis* strain PCM2 with 0.03 μm AmB showed no different RDC values, indicating the same number of chromosomal copies (copy) per haploid genome when compared between the genomes of this parasite under the AmB-treated and AmB-free conditions. Chromosomes 1 (1.31, 1.34), 2 (1.17, 1.19), 10 (1.45, 1.40), 29 (1.27, 1.26) and 31 (1.80, 1.79) appeared to be more than one copy due to the RDC values of greater than 1.0 from control condition and AmB treated condition, respectively (**Fig 1** Left panel). Only coding sequences (CDSs) were used to calculate the RDC values to increase the accuracy and reduce noise background data. This method reduced interference from repeat sequences in the genome, which makes RDC values higher than calculating from coding sequences (**Fig 1** Right panel). The RDC values inferred from only CDSs brought most chromosome sets closer to one except for those of chromosomes 29 (1.42, 1.41) and 31 (1.93, 1.92).

For the chromosome somy estimation, allele frequency was used for calculating the distribution of allele frequency ratios of *L*. *orientalis* strain PCM2 in the heterozygous position in the AmB-free control (**S2A Fig**) and the AmB-treated group (**S2B Fig**). The obtained allele frequencies were close to the calculated RDC values. The distribution of the allele frequencies on each chromosome was computed. Most chromosome allele frequency ratios were 0.5, indicating that most of the chromosomes were diploid. In contrast, chromosomes 29 and 31 had chromosome allele frequencies of approximately 0.33 and 0.66, representing chromosomes in a trisomy state, consistent with the calculated RCDs.

**Fig 2:**
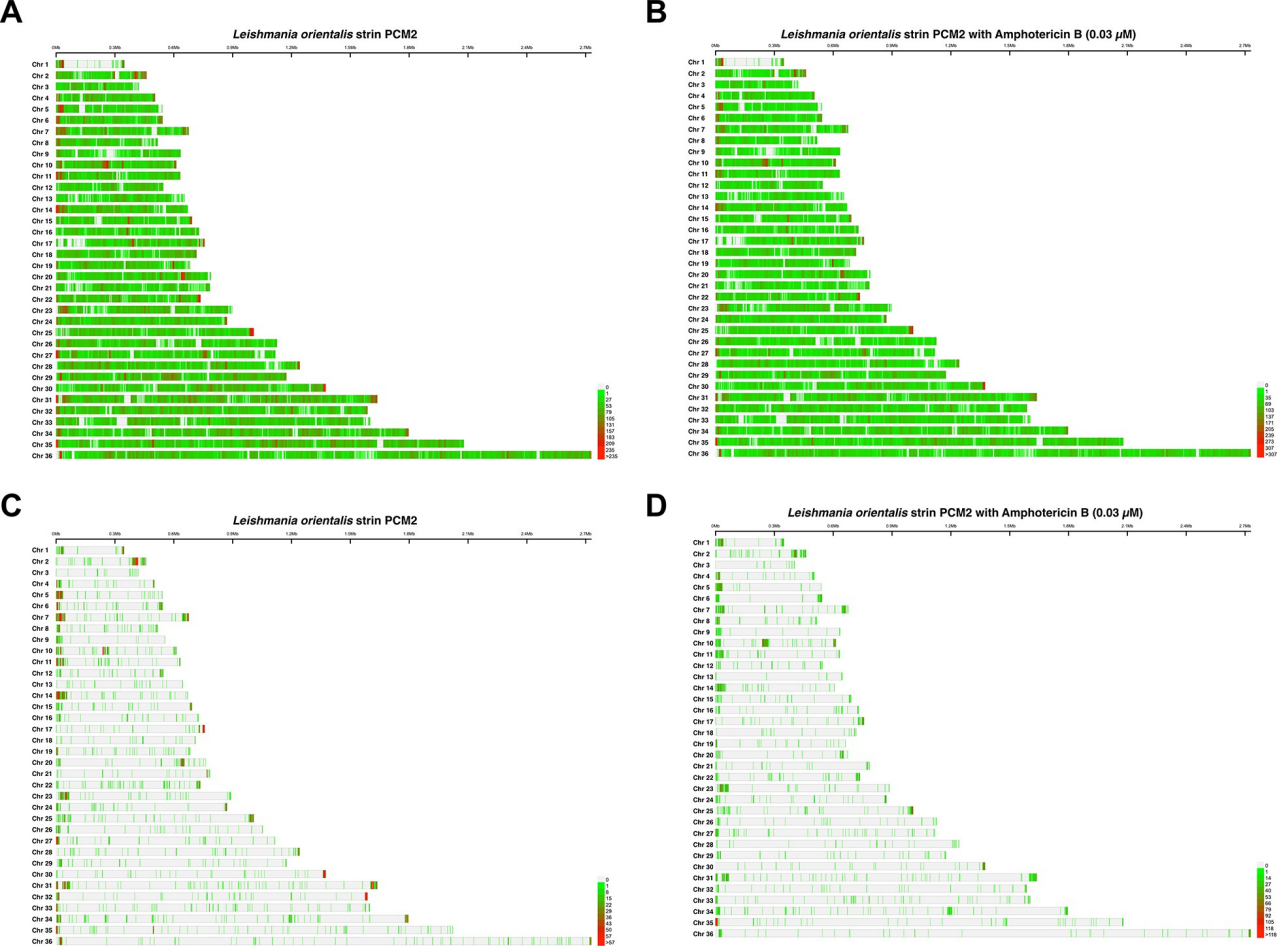
Comparative analysis of single nucleotide polymorphisms (SNPs) distribution across the chromosomes of *L*. *orientalis* strain PCM2 cultured in the Amphotericin B-free control (A) and the Amphotericin B-treated group (B). Unique SNPs emerging solely within the control (C) or treatment (D) groups were selectively presented. The x-axis showed nucleotide positions summarised as the bin size of 10,000 bp, and the y-axis represented the *L*. *orientalis* chromosomes. The colour scale indicated the graph’s SNP density per bin size: red for the high number of SNPs and green for the low number of SNPs per bin.

The analysis of SNP distribution patterns of *L*. *orientalis* after exposure to the AmB treatment revealed substantial variability at the SNP loci. There were 157,681 SNP positions in the control group (**Fig 2A**) and 157,306 positions in the AmB-treated group (**Fig 2B**). Within these SNP variations, condition-specific SNPs were observed, including 4,551 SNPs (35.54% Upstream, 37.31% Downstream, 17.66% Intergenic, 0.44% Intron, and 6.62% Exon) found only in the AmB-free control group (**Fig 2C**) and 4,221 SNPs (33.38% Upstream, 37.95% Downstream, 15.86% Intergenic, 0.65% Intron, and 8.38% Exon) in the AmB-treated group (**Fig 2D**). As displayed in Fig 2, the high number of SNPs per 10,000 bp preferentially located towards the ends of the chromosome, and certain remained in between the chromosomes.

**Fig 3:**
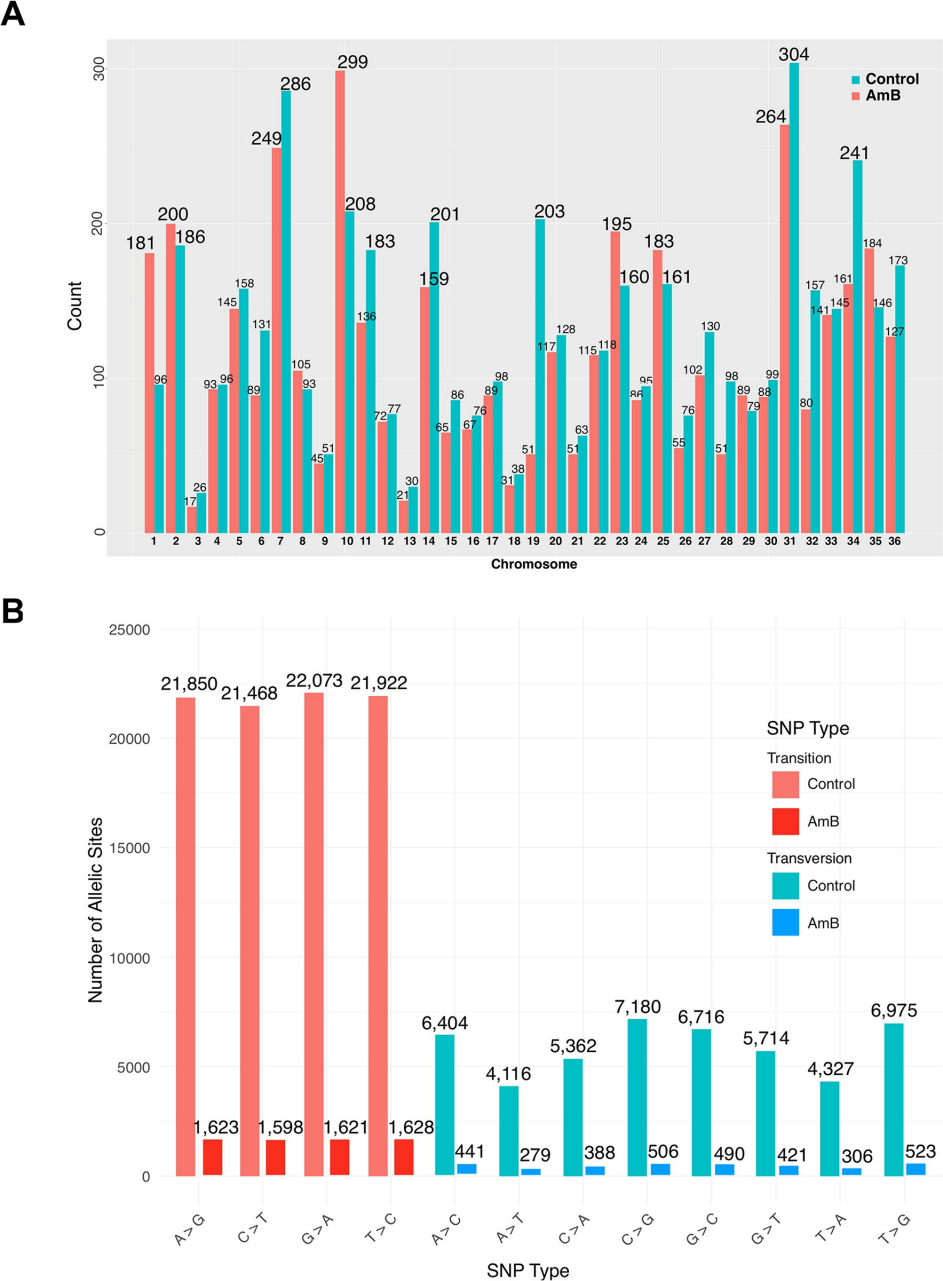
Comparison of the single nucleotide polymorphism (SNPs) number across the chromosomal landscape of *L*. *orientalis* strain PCM2 under the Amphotericin B-treated (red) and control (green) groups. The x-axis indicated the chromosome number, and the y-axis showed the number of SNPs. The SNP number of each chromosome was labelled on the bar graph (A). The altered distribution of SNPs across the genome was categorised by types of base substitutions (transition and transversion) on the Y-axis. Each point on the X-axis represented the count of base substitutions at specific allelic sites. Different colours indicated distinct allelic types or categories, visually representing the frequency and distribution of SNPs (B).

In addition, the number of SNPs per chromosome of *L*. *orientalis* strain PCM2 from the AmB-free control and AmB-treated groups was compared (**Fig 3A**). The SNP number across individual chromosomes exhibited a notable variability. A higher SNP number was observed on most chromosomes (3–9, 11–22, 24, 26–28, 30–34, 36, and notably chromosome 19) from the AmB-free control group compared to the treatment group. The comparison also underscored interesting evidence of higher SNP numbers on seven chromosomes (1, 2, 8, 10, 23, 25, 29, and 35) from the AmB-treated *L*. *orientalis* strain PCM2 genomes. The SNP number of chromosomes 1 and 10 was exceptionally high for the AmB-treated group compared to the control. Specifically, chromosome 1 exhibited 96 and 181 SNPs, and chromosome 10 exhibited 208 and 299 SNPs in the control and AmB-treated groups, respectively. The SNP variations can be categorised into transitions and transversions. The most prevalent SNP type in both conditions is transition, with G to A transitions occurring 22,073 times in the control group and T to C transitions occurring 1,628 times in the AmB-treated group. Additionally, transversions, specifically C to G, were observed 7,180 times in the control group and 506 times in the AmB-treated group (**Fig 3B**).

**Fig 4:**
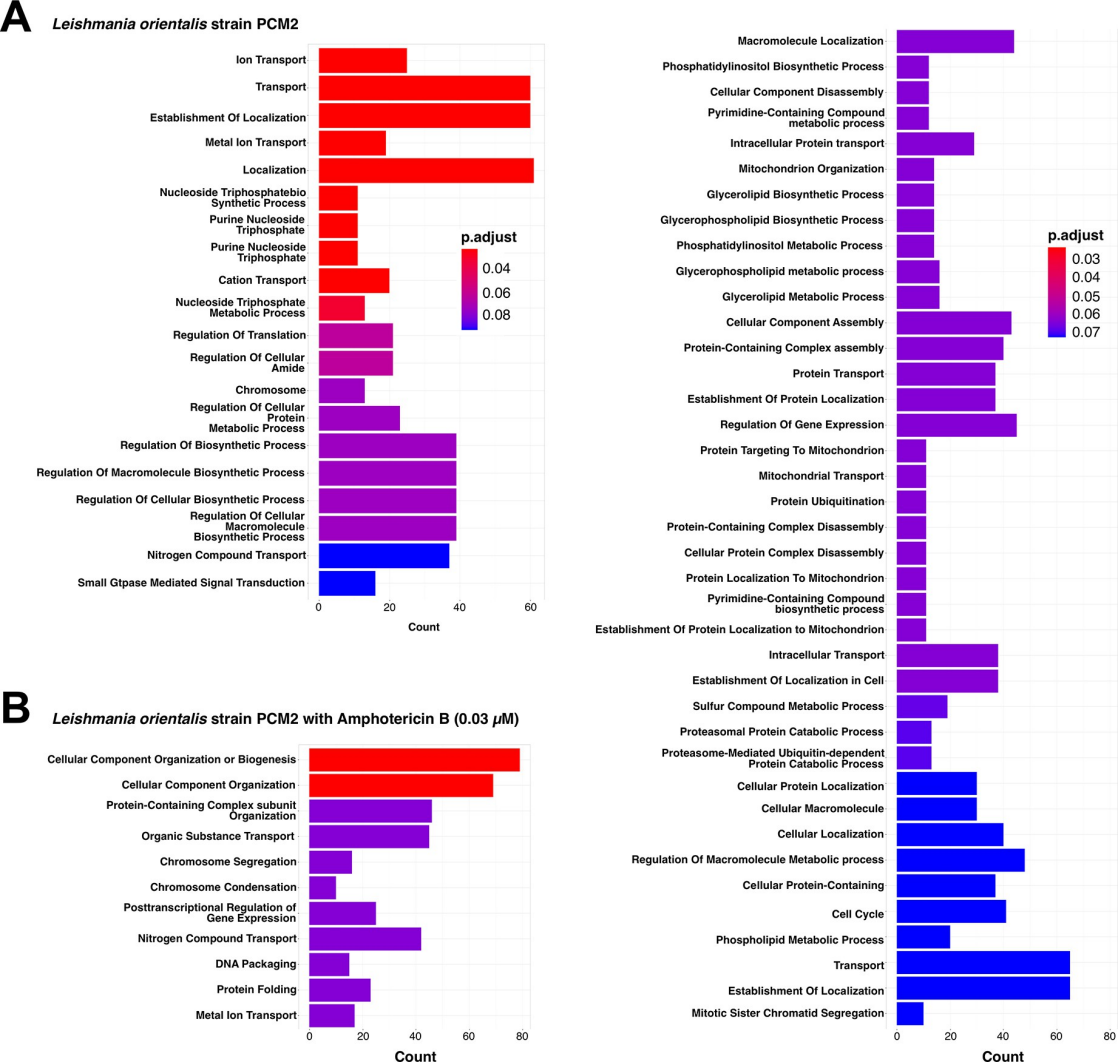
Enrichment analysis of distinct single nucleotide polymorphisms (SNPs) categorised according to the gene ontology’s biological process (BP) in *L*. *orientalis* strain PCM2. The analysis encompassed both the normal condition (A) and the condition treated with Amphotericin B (AmB) (B lower left and right graphs). The colouration of enriched terms corresponded to their adjusted *p*-values, thereby delineating their statistical significance.

**Fig 5:**
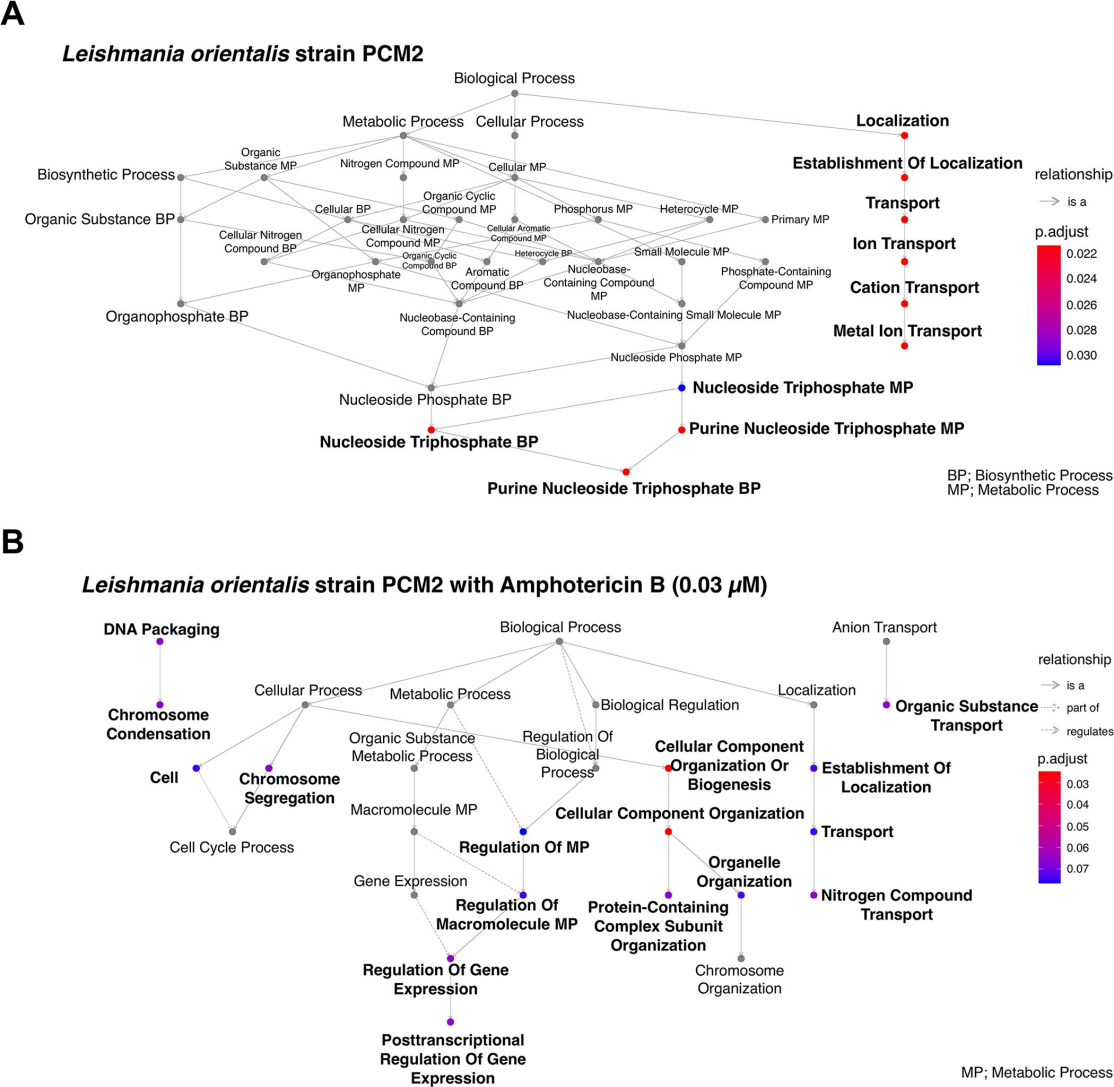
Enrichment map analysis of distinct single nucleotide polymorphisms (SNPs) categorised according to the gene ontology’s biological process (BP) in *L*. *orientalis* strain PCM2. The analysis included SNP sets unique to the normal condition (A) and those exclusive to the Amphotericin B-treated condition (B). The colour gradient from blue to red indicates the significance of the enriched terms, and the connections illustrate the relationships between gene sets in each condition.

The genomic landscape of single nucleotide polymorphisms (SNPs) within the control group revealed a notable distribution across diverse pathways within the biological process category. A significant proportion of these SNPs was distinctly associated with genes involved in transporter functions, particularly cation and metal ion transporters, as well as in the synthesis of genetic precursors, such as purine nucleoside triphosphate biosynthesis (**Figs 4A and 5A**). In contrast, the SNP profile within the *L*. *orientalis* strain PCM2 genome subjected to AmB treatment at 0.03 μM exhibited a greater diversity of pathways (**Fig 4B**). These enriched SNPs showed notable involvement in a broad range of pathways central to genetic and cellular regulation. Specifically, they were engaged in DNA conformational dynamics, including DNA packaging and chromosome condensation, cell cycle dynamics, gene regulation, organisation of cellular components, nitrogen metabolic pathways, and the transport of organic substances (**Fig 5B**).

### 3.2 Comparative transcriptome of *L*. *orientalis* strain PCM2 treated and non-treated with Amphotericin B

**Fig 6:**
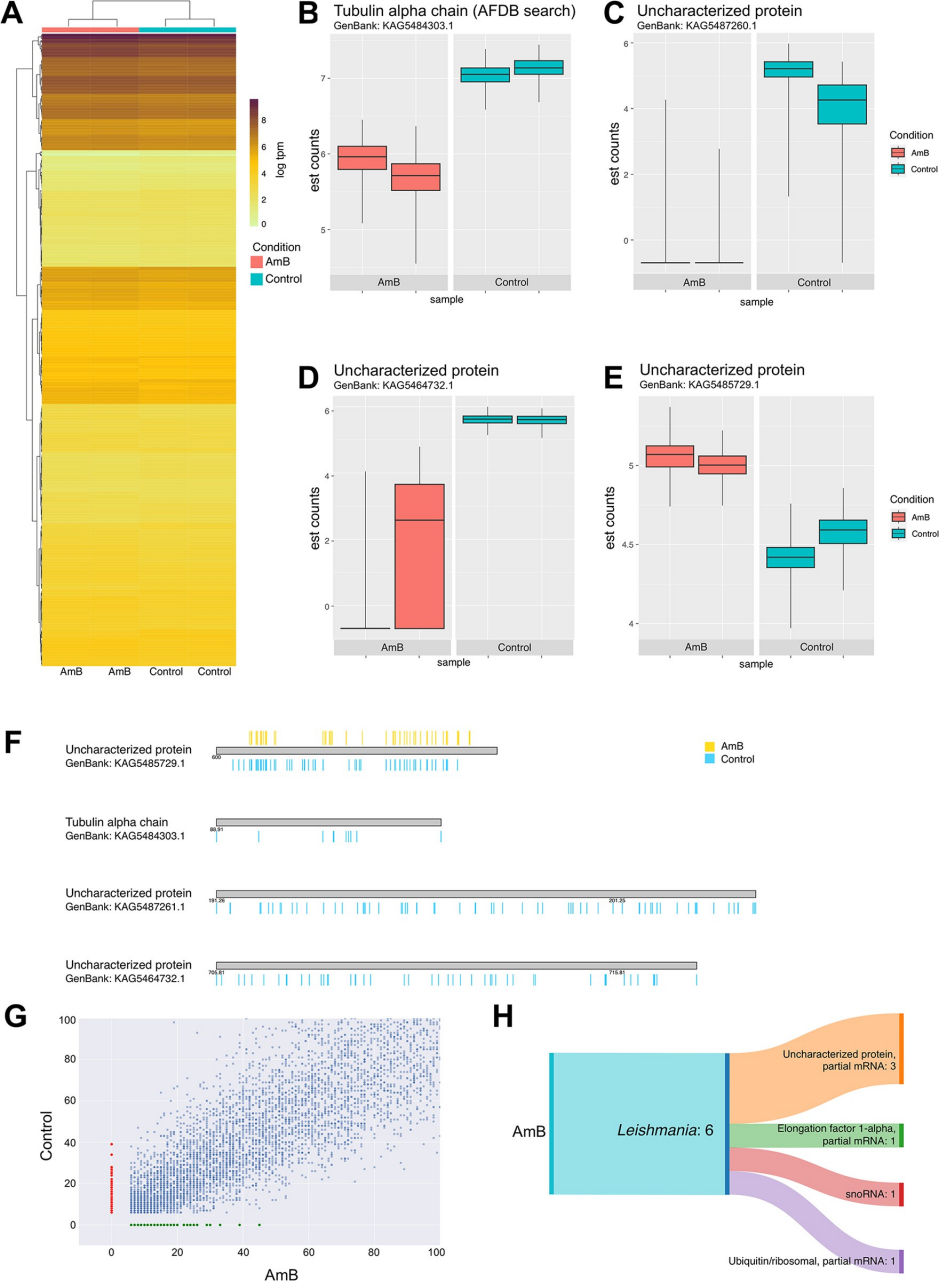
Comparative transcriptome analysis of differential gene expression of *L*. *orientalis* strain PCM2 under the Amphotericin B-free control (depicted in blue) and Amphotericin B-treated (depicted in red) conditions. The differential expression of genes was evaluated through the Wald test and subjected to a q-value threshold of ≤ 0.5 (A). The colour gradient reflected the logarithmic mean of transcripts per million (TPM). Genes counts were estimated and compared between the two conditions, including tubulin alpha chain (B) and three uncharacterised proteins (C-E). The x-axis of the box plots showed experimental conditions, and the y-axis indicated estimated counts (est count). (F) The figure illustrated the distribution of single nucleotide polymorphisms (SNPs) within four specific genes in the AmB-treated group. The x-axis represented nucleotide positions aggregated into bins of 100 base pairs. The colour scale on the graph indicated the SNP density per bin size, with red indicating a high density of SNPs within a bin and green indicating a low density of SNPs within a bin. The scatter plot from the k-mer analysis identified unique read sequences exclusive to either the control (red dot) or AmB treatment (green dot) groups, highlighting potential control or AmB-specific transcripts or regulatory elements (G). Annotation of specific genes related to the AmB treatment condition using BLASTn classified into six candidate genes and represented in SankeyMATIC illustrator, highlighting potential cooperative role contributing to the observed response to AmB treatment (H).

Principal component analysis (PCA) was used to analyse sample variation and demonstrated the capacity to identify and address outliers (**S3 Fig**). The differential gene expression analysis of 8,158 genes of *L*. *orientalis* strain PCM2 under the growth in the presence and absence of Amphotericin B identified the expression of 673 genes significantly different between the two conditions. The beta value was estimated from the regression coefficient using maximum likelihood estimation (MLE), which embodied the logarithmic representation of fold changes (log2) occurring between conditions. Differential gene expression was assessed using the Wald test, with significance determined by a q-value threshold of ≤ 0.5 (**Fig 6A**). In particular, the range of gene expressional changes was broad from 5-fold increased to subtle -0.05-fold alteration. Four genes exhibited a high level of fold change values including tubulin alpha chain (GenBank; KAG5484303.1, 1.25 fold change) (**Fig 6B**), three uncharacterised proteins (KAG5487260.1, 5.45 fold change; KAG5464732.1, 4.70 fold change; and KAG5485729.1, -0.53 fold change) (**Fig 6C–6E**). Intriguingly, it became evident that most of these four genes displayed a discernible downregulation pattern upon exposure to the AmB treatment (**Fig 6B–6D**). Specifically, the tubulin alpha chain gene (**Fig 6B**), a pivotal player in cellular structural dynamics, was observed to undergo a noteworthy downregulation by a factor of 1.25, as underpinned by the estimations of mean counts, with an estimated count of 6.48. This transcriptional modulation highlighted the potential impact of AmB treatment on the intricate machinery of microtubule dynamics, indicating potential clinical and mechanistic significance.

SNPs were distributed within the upstream regions across all four genes, and the results showed that the number of SNPs was reduced or disappeared in the AmB-treated group. (**Fig 6F**). Intriguingly, a noteworthy observation arose from the absence of detected SNPs within the transcribed RNA sequences. The k-mer analysis revealed unique read sequences exclusive to the control or AmB treatment group (**Fig 6G**). The comprehensive annotation approach was undertaken using Trinity and Blastn against the NCBI RNA database to identify the functional significance of these distinctive sequences. The resulting annotations underwent stringent refinement, wherein only sequences exhibiting a percentage of identity and coverage greater than 80% were retained for subsequent analyses. In addition, a notable proportion of the contigs persisted in an unannotated or uncharacterised, with 34.17% in the control group and 34.91% in the AmB-treated group, indicating the potential existence of novel transcripts or regulatory elements. A predominant subset of the annotated contigs in the control group comprised partial mRNA sequences (32.58%), including the 60S, 40S, and 28S subunits. Conversely, in the AmB-treated group, the annotation revealed the presence of four distinct gene groups, including uncharacterised proteins, elongation factor 1-a, small nucleolar RNA (snoRNA), and ubiquitin/ribosomal mRNA (**Fig 6H**).

**Fig 7:**
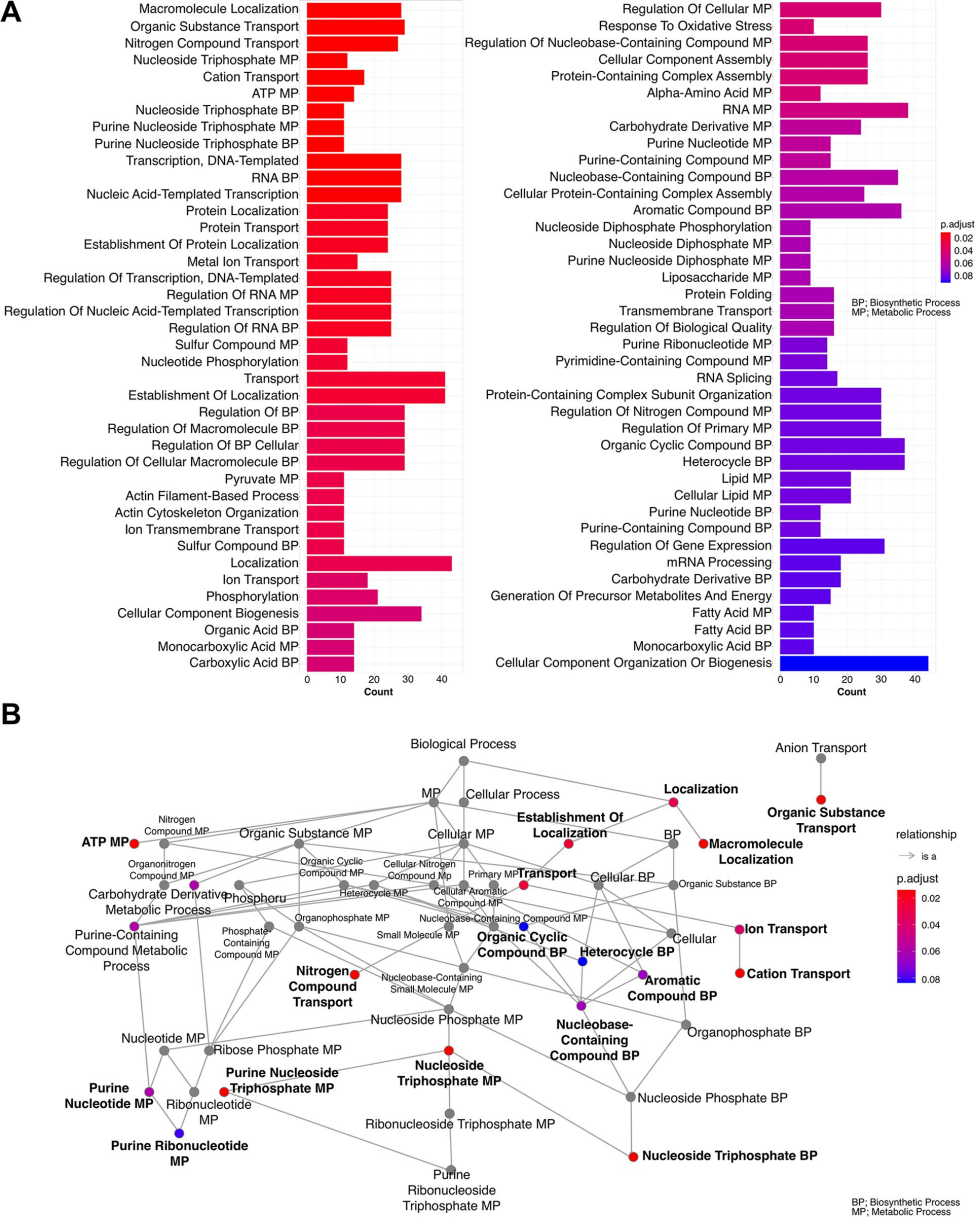
Functional enrichment of gene ontology (GO) in the biological process (BP) category assigned to the differentially expressed genes from the comparison between *L*. *orientalis* strain PCM2 growth in the control and the AmB-treated conditions (A). The colour gradient represented the adjusted *p*-values. Hierarchical clustering employed Jaccard’s similarity index (J.C.) and Ward’s method to enrich the GO terms and labelled them with high-frequency similarity (B). An enrichment map presented enriched terms as a network by interconnecting overlapping gene sets. This visualisation enhanced the identification of functional modules arising from mutual gene sets.

**Fig 8:**
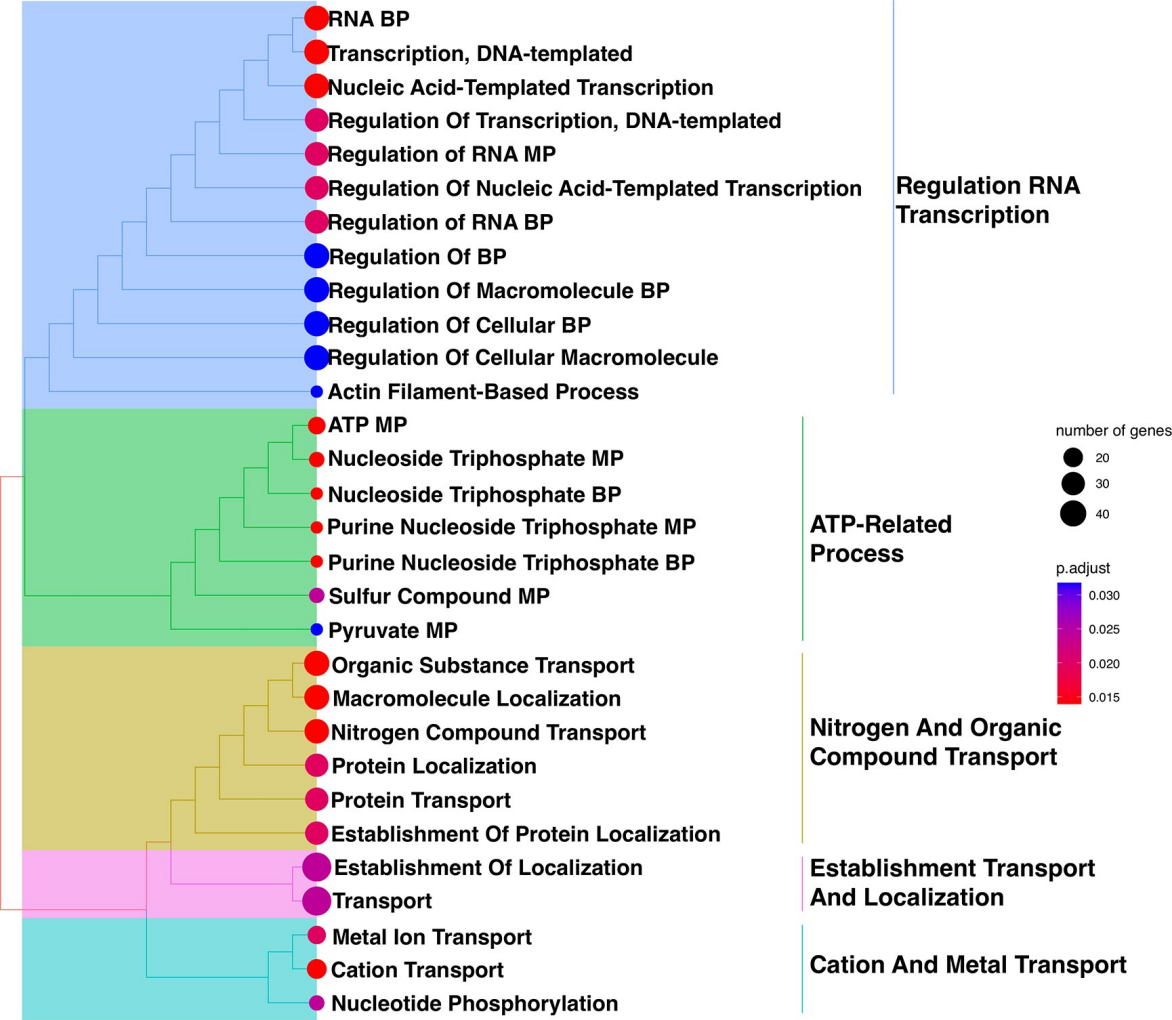
The hierarchical clustering of the enriched GO terms derived from significant differential expressed genes of *L*. *orientalis* strain PCM2 under the growth in the AmB-treated and control conditions. The clustering was based on pairwise similarities of enriched terms calculated using the Jaccard’s similarity index (JC). The function segmented the tree into five subtrees and labelled these subtrees with high-frequency words, and the radius of circular dots represented the gene number. The *p*-values were adjusted and classified from low (red) to high (blue), providing a visual representation of the significance of each term.

After identifying 673 genes exhibiting statistically significant alterations in expression levels, a comprehensive exploration of their functional implications was performed by assigning Gene Ontology (GO) terms under the biological process category (**Fig 7A and 7B**). The most highly significant GO terms were related to localisation, transportation, and transcription regulation (**Fig 7A**). This intricate analysis revealed a notable convergence between this gene subset and the genes with 737 SNPs of the AmB-treated group (**Fig 5B**), which highlighted significant changes of genes in the transport pathway of organic substance and nitrogen compound within this treatment group (**Fig 7B**). The enriched GO term clustering of the differentially expressed genes identified five subtrees of the main functions including regulation RNA transcription, ATP-related process, nitrogen and organic compound transport, establishment transport and localisation, and cation and metal transport (**Fig 8**). These five clusters highlighted the importance of gene alterations in gene expressional regulation, energy metabolism, nucleotide synthesis, cellular transport mechanisms, and protein localisation.

**Fig 9:**
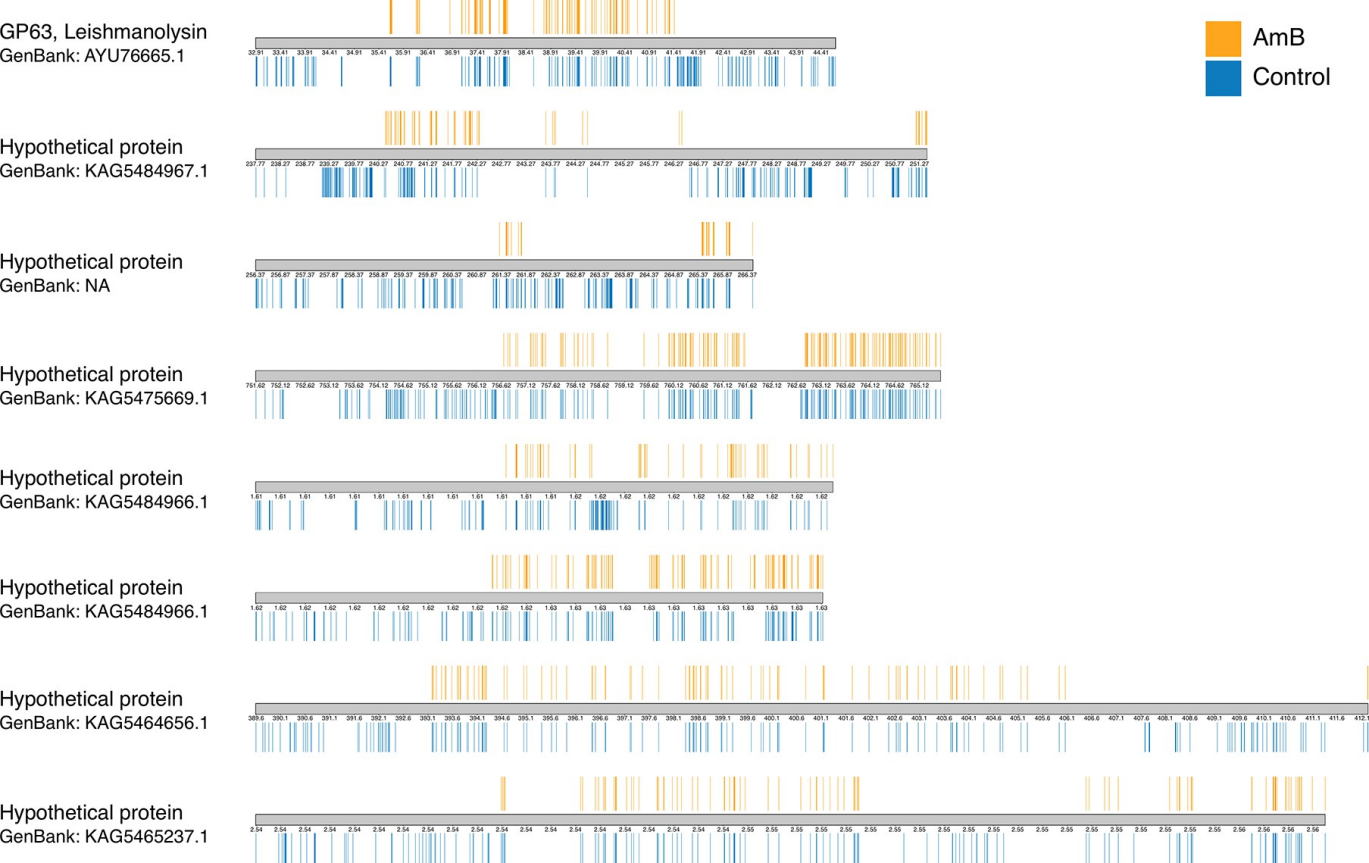
SNPs distribution and density changes of selected regions in the *L*. *orientalis* strain PCM2 genome. This karyotype plot illustrated the distribution and density of SNPs in the upstream regions of the first eight genes with the highest SNP density. The plot compared SNPs between two conditions: normal (bottom panel, blue) and Amphotericin B-treated (top panel, orange).

The first eight genes with the highest SNP distribution were selected, and the variations in their upstream regions were visualised. These genes were GP63, Leishmanolysin protein (GenBank: AYU76665.1), and seven hypothetical proteins. The visualisation highlighted the variability in SNP distribution, revealing significant differences between the control and AmB-treated groups. These differences underscored the presence of SNP hotspots in the upstream regions of each gene (**Fig 9**). Furthermore, the SNP distribution was categorised into unique sites and shared sites (**Table 1**) and showed the presence of distinct SNPS at different positions of these eight genes, where the number of SNPs in the control group was higher than in the AmB-treated group. Both groups showed unique SNPs specific to each group, except for genes with the accession number of KAG5464656.1 and KAG5465237.1, where all SNPs in the AmB-treated group appeared in the control group. The results confirmed a decrease in SNPs in the AmB-treated group compared to the control group. Additionally, novel SNP loci specific to each condition demonstrated the distinct genetic changes induced by the AmB treatment. This analysis emphasises the importance of understanding SNP distribution and its implications for gene function and drug response.

### 3.3 Absence of *Leishmania* RNA viruses 1 and 2 in the genome of *L*. *orientalis* strain PCM2

The LRV was analysed by blasting the *L*. *orientalis* strain PCM2 transcripts to the local LRV reference sequence database. Results showed no significant matches. *De novo* assembled contigs were blasted against the local LRV database to confirm the absence. This comprehensive approach also returned no positive hits. The results proved no detectable LRV1 and LRV2 sequences in the *L*. *orientalis* strain PCM2 RNA data.

**Table 1:** SNP distribution in control and AmB-treated conditions.

Protein Name	GenBank Accession Number	Number of SNPs		Number of Shared SNP Location
		Control (Unique SNPs)	AmB-treated (Unique SNPs)	
GP63, Leishmanolysin	AYU76665.1	226 (130)	117 (21)	96
Hypothetical protein	KAG5484967.1	237 (202)	65 (30)	35
Hypothetical protein	NA	212 (179)	36 (3)	33
Hypothetical protein	KAG5475669.1	243 (86)	159 (2)	157
Hypothetical protein	KAG5484966.1	158 (105)	62 (9)	53
Hypothetical protein	CBZ24361.1	159 (56)	124 (21)	103
Hypothetical protein	KAG5464656.1	152 (70)	82 (0)	82
Hypothetical protein	KAG5465237.1	152 (60)	92 (0)	92

* The numbers in parentheses were the number of unique SNPs.

## 4. Discussion

*Leishmania* parasites demonstrate a remarkable capacity to withstand substantial aneuploidy, encompassing variations in chromosomal "somy" such as disomy, trisomy, and tetrasomy [9,58]. Previous studies indicated a potential correlation between aneuploidy or gene copy number variation (CNV) and heightened expression levels of specific genes implicated in drug resistance mechanisms [11,59]. Amphotericin B (AmB) is employed as the preferred pharmacological intervention for acquired drug resistance arising from conventional antimony-based therapy. AmB is used in regions with endemic visceral leishmaniasis, such as Bihar, where a substantial proportion of approximately 64% of cases currently manifested resistance to antimonial agents [22], or becomes an option for treating the visceral leishmaniasis with HIV co-infection [60]. The use of a sub-lethal concentration of AmB that was ten times lower than the IC50 in 72 hours was optimal for studying the responses and adaptations of organisms to stressors in a manner that represented the real-world conditions and ensured that the majority of the population under study was not completely eliminated [61–63]. The detection of genetic variations under such circumstances became pivotal, as it provided a unique insight into genetic adaptations that promoted survival traits under these suboptimal conditions. Moreover, Iantorno et al. [64] conducted a comprehensive investigation by integrating genome-wide high-throughput DNA sequencing and RNA-seq analysis. They revealed significant correlations between chromosome aneuploidy (somy), CNVs, and SNPs across 14 *Leishmania tropica* isolates in the promastigote stage under *in vitro* growth conditions. Their study demonstrated that alterations in the gene dosage, at both individual gene and chromosomal "somy" levels, accounted for more than 85% of the total variation in gene expression, particularly for genes exhibiting a two-fold or greater change in expression. For confirmation, *Leishmania tropica* isolate Ackerman, which had trisomic on chromosome 6 and some part of chromosome 11 was trisomic, had a higher expression level of genes on these chromosomes compared to isolate K26 [64]. Conversely, chromosome 21 of the isolate K26 was trisomic compared to the isolate Ackerman, and the K26 had a higher expression level of the genes on this chromosome. However, the application of AmB at concentrations 10-fold lower than the IC50 value might not attain a threshold necessary for eliciting alterations in the level of chromosome copy numbers under a short period (**Fig 1**). Previous investigations demonstrated that the cell cycle of kinetoplasts exhibited interspecies variation: for instance, the *in vitro* promastigote cell cycle of *L*. *mexicana* was approximately 7.1 hours [65], while that of promastigotes from *L*. *major* extended to 10.2 hours [66]. Similarly, *Trypanosoma brucei* manifested a cell cycle duration of approximately nine hours [67]. The varied cell cycle times implied that over 72 hours, the *in vitro* promastigotes of *L*. *orientalis* strain PCM2 could have potentially undergone 7 to 10 cell cycles, suggesting the possible proliferation of the parasitic cell populations harbouring and accumulating SNPs within their chromosomes [68]. The previous study using fluorescence *in situ* hybridisation (FISH) to determine the number of homologous chromosomes per individual cell in *Leishmania major* during interphase and mitosis revealed that *Leishmania* chromosomes could exist in at least two distinct ploidy states (monosomic, disomic, or trisomic) within the cell population, leading to considerable chromosomal mosaicism and changing in every generation [69]. These previous results were consistent with the present study, showing the occurrence of disomic and trisomic stages in the *L*. *orientalis* cells.

The variation of SNP distribution on the genome might influence the intricate regulatory interplay that governs genomic variation in *Leishmania* (**Fig 2**). The regulatory interplay in *Leishmania* is a complex system that involves various genetic elements, including SNPs which can affect gene expression, protein function, and other molecular processes. Understanding the relationship between SNP distribution and regulatory mechanisms is crucial for unravelling the genomic variations observed in *Leishmania* parasites. The intricate regulatory interplay in *Leishmania* involves multiple layers of gene regulation, including transcriptional control, post-transcriptional modifications, and epigenetic regulation. SNPs can disrupt these regulatory processes by altering DNA sequences or affecting the binding of transcription factors and other regulatory proteins. Consequently, changes in SNP distribution may lead to variations in gene expression patterns and contribute to the observed genomic diversity in *Leishmania*. The heightened SNP frequencies within the control group of the AmB-free condition (**Fig 3**) suggested potential genomic adjustments specific to this condition as frequent appearance of SNPs at the up- or down-stream and intergenic regions. The exceptions observed within specific chromosomes could arise from various factors, including localised genetic determinants, structural features, or other contextual influences [64]. The observed fluctuation in SNP distribution across chromosomes could underscore the intricate interplay of genetic variation and regulatory dynamics in response to the presence and absence of AmB. Furthermore, several studies suggested that SNPs in non-coding regions could influence transcription factor binding sites, thereby altering gene expression. Unlike typical eukaryotic cells, trypanosomatids undergo polycistronic transcription, where multiple genes are transcribed into a single RNA molecule. Although gene regulation in *Trypanosoma* primarily depends on post-transcriptional mechanisms, variations in non-coding regions may also regulate the gene expression [4,70–72]. Several studies suggested that SNPs in non-coding regions could influence the transcription factor binding sites, thereby altering human gene expression [73–75]. Therefore, *L*. *orientalis* might regulate responses towards the AmB stress by the SNP alteration of regulatory regions.

Notably, these enriched SNPs exhibited pronounced involvement in diverse facets of genetic and cellular regulations during the AmB treatment (**Figs 4B and 5B**). This spectrum encompassed pathways centred on DNA conformation dynamics, prominently manifesting under DNA packaging and chromosome condensation processes (AAA+ ATPase domain-containing protein) might play a role in gene exchange through extracellular vesicles [76] and proteasomal degradation [77–79]. Such observations implicated a potential link between these genetic variations and the orchestration of higher-order chromatin structures, suggesting potential repercussions on gene expression and genome stability. Previous findings highlighted that within the AmB-resistant *Leishmania* promastigote, there was a substitution of ergosterol by a precursor molecule cholesta-5,7,24-trien-3β-ol [20,23], accompanied by an elevation in both the AmB efflux mechanism and the machinery responsible for scavenging reactive oxygen species (ROS) [23]. Because AmB functions through its attachment to ergosterol, a sterol found within the membranes of both fungi and *Leishmania* parasites, this interaction results in the disturbance of the cell membrane’s structural integrity. Consequently, the membrane becomes more permeable, causing essential cellular components to leak. It is noteworthy that while ergosterol is the primary focus of this interaction, additional constituents of the membrane, such as ion channels and transporters, nitrogen-containing compounds transport, including amino acids, are essential for various cellular functions, such as protein synthesis, nucleotide synthesis, and energy production. These functions could also experience impacts from the cell membrane disruption, such as influence on the transport of nutrients and metabolites, potentially impacting the availability of nitrogen-containing compounds [80]. This process could potentially lead to the leakage of ions like potassium ions (K+) and contribute to the eventual demise of the cell [23].

Downregulation of tubulin alpha gene expression (**Fig 6B**) could be seen in previous *Leishmania* studies [81]. The extensively conserved alpha and beta-tubulin subunits engage to form α/β-tubulin heterodimers, which constitute fundamental constituents of the eukaryotic cytoskeleton. These heterodimers contribute to essential cellular processes such as cell division machinery, intracellular transport, and the motility of ciliary and flagellar structures [82]. The synthesis of tubulin demonstrates a correlation with morphological alterations occurring during the transition from promastigote to amastigote stages. Notably, promastigotes exhibited higher tubulin synthesis than amastigotes [83]. Previous research indicated that the reduction in mRNA levels of α-tubulin observed under promastigote culture conditions at 35°C was concomitant with changes in parasite morphology [84,85]. The *L*. *donovani* with arsenite resistance showed downregulation of tubulin protein expression, demonstrating heightened susceptibility to paclitaxel compared to the wild-type strain. In arsenite-resistant *L*. *donovani* promastigotes exposed to paclitaxel treatment, the levels of β-tubulin exhibited an approximate increment of 40%. The α-tubulin exhibited a reduction of 26% in expression within the resistant strain and an elevation of 35% in the wild-type strain [86,87]. If the AmB effects lead to alterations in cellular components or membrane properties, it could indirectly affect microtubule dynamics and potentially impact cell division and other cellular functions [88]. In addition, microtubules are also a drug target that has been studied in various parasites, including *Leishmania* [81,89–91]. While direct evidence of the relationship between the tubulin alpha chain and AmB is limited, there is potential for the AmB actions to influence the microtubule dynamics and cellular processes indirectly. The other groups of genes that changed their expression levels have not yet identified their functions (**Fig 6C–6E**), and further research could be conducted to elucidate the functions of these hypothetical proteins. AmB disrupts *Leishmania* membrane and induces the oxidative street within the cells [92]. The oxidative stress response involves various pathways and molecules (**Fig 6H**), including elongation factor 1-α (EF1-α), which plays a critical role in protein biosynthesis and has been reported as a *Leishmania* virulence factor [93] and has been implicated in *Leishmania*’s response to nutritional stress [94]. Recent studies suggested that EF1-α could be a promising candidate for drug targeting [95] and vaccine development [96,97]. However, its specific role in oxidative stress response mechanisms has not yet been thoroughly investigated. While small nucleolar RNAs (snoRNAs) are crucial for rRNA processing in *Leishmania*, their expression and function changes can help the parasite adapt to environments [98,99]. By modifying ribosomal function, snoRNAs optimize the translation process under stress conditions, supporting the parasite’s survival [100].

The kinetoplasts’ unique genome structure is characterised by polycistronic gene clusters and the absence of promoter-mediated regulation that undergoes constitutive transcription into mRNA molecules. The post-transcriptional mechanisms act as primary regulatory axes in expression levels [4,101–104]. Nonetheless, our investigation revealed a convergence of certain biological processes in which shared occurrences of SNPs (**Fig 7**) and the altered gene expression were identified within the same biological process group, such as nitrogen compound transport and organic substance transport (**Fig 7B**). Another set of biological process categories was identified, encompassing ATP metabolic processes and nucleoside processes (**Figs 7 and 8**). Alterations in the genes associated with ATP metabolic pathways were reported in the previous study, such as the combination treatments of drugs such as amphotericin B, miltefosine, paromomycin, and SbIII. Observing the development of resistance over ten weeks in *Leishmania donovani* revealed that the resistant parasites had altered levels of thiol, ATP, and mitochondrial membrane potential. Importantly, resistance to one drug combination led to cross-resistance to other anti-leishmanial drugs [105]. In addition, *L*. *infantum* AmB resistance showed that the H1A-2 P-type H^+^-ATPase was down-regulated. Overexpression experiments suggested that this gene alone may not be an AmB resistance gene because it could still contribute to AmB resistance in terms of proton activity, leading to futile ATP consumption in the presence of membrane pores and unnecessarily depleting the parasite of ATP [24]. Another study evaluated the effect of resveratrol in *Leishmania amazonensis*. Treatment with resveratrol increased the percentage of promastigotes in the sub-G0/G1 phase of the cell cycle and reduced the mitochondrial potential [106]. These phenomena served as the drug-resistance mechanism, safeguarding against ATP depletion and preserving mitochondrial membrane potential from drug-induced effects [105,106].

In addition, previous studies also observed changes in gene expression in antimony-resistant *Leishmania* at the chromosome level. Further analyses, such as Southern blots and comparative genomic hybridisations, indicated that this was due to either the presence of supernumerary chromosomes or the loss of a chromosome, suggesting a potential connection between aneuploidy and drug resistance in *L*. *infantum* [107]. On the other hand, the results of this study indicated the absence of detectable alterations at the chromosome level (**Fig 1**) and in gene copy numbers (**S1 Table**) following AmB treatment at a concentration ten times lower than the IC50. This lower concentration may be below the stress threshold required to induce chromosome-level changes. Other research used concentrations in the IC50 range for drug treatment experiments [25] or resistant *Leishmania* strains [20,24]. This study provided a clue to understanding the intricate interplay between genetic variations and gene expression dynamics. The implications of the potential role of DNA variants in the regulation of gene expression in *Leishmania* are multifarious. Genetic variants within regulatory regions may exert a cascading impact on gene expression profiles, thereby influencing cellular phenotype and function in other species [108]. The intricate nature of gene regulation within *Leishmania*, characterised by multifaceted regulatory tiers including post-transcriptional and post-translational processes, is mirrored in the observed gene expression alterations [72]. In this research, our hypothesis posits that the mechanisms of gene expression regulation by SNPs might affect the mRNA stability varying by type of SNPs and position, as shown in other organisms’ studies [109–111]. This mechanism has not been thoroughly studied in *Leishmania*. The observed associations underscored the complex interplay between genetic variations and cellular functionality, thus inviting further investigation into the mechanistic underpinnings of these intricate relationships.

Lastly, the manifestation of *Leishmania* RNA virus (LRV) within the *Leishmania* parasite conferred discernible advantages to the context of leishmaniasis, particularly concerning parasite persistence and augmentation in disease susceptibility [56] and related with treatment failure [112,113]. Reports showed that LRVs might be associated with drug resistance, such as in *L*. *naiffi* with pentamidine resistance [114] and *L*. *major* with meglumine antimoniate resistance [115]. The absence of LRVs in this investigation may make *L*. *orientalis* strain PCM2 susceptible to the six antileishmanial drugs, including AmB [56]. The symbiotic relationship between LRVs and *Leishmania* intricately influences the pathogenicity dynamics, demonstrating a multifaceted interplay that extends beyond a mere parasitic-host relationship. LRV’s role in enhancing the persistence of the *Leishmania* parasite within its host represents a critical aspect of the pathophysiological landscape, facilitating sustained parasitic survival and prolonged infection. Furthermore, the nuanced interactions orchestrated by LRVs contribute to an intricate modulation of the host immune response, thereby amplifying the susceptibility to leishmaniasis. These associations underscore the significance of comprehensive investigating which offers valuable insights into the molecular mechanisms underlying the progression and outcomes of leishmaniasis.

## 5. Conclusions

In conclusion, the findings of this study provided valuable insights into the interplay between genetic variations and gene expression dynamics. Identifying gene clusters within the shared biological processes highlighted a level of coordinated regulatory dynamics that transcended individual genes. This sheded light on the orchestration of cellular responses to the AmB exposure. The potential implications of DNA variants in regulating gene expression in *Leishmania* are wide-ranging. Genetic variants within the regulatory regions could trigger cascading effects on the gene expression profiles, thereby influencing cellular phenotypes and functions. Moreover, this study served as a foundation for future research endeavours to decipher the nuances of genetic regulation in the context of cellular responses to the AmB exposure of *Leishmania*, ultimately advancing our comprehension of the broader interconnections between genetic variation and cellular dynamics. However, these findings were preliminary, urging further comprehensive investigations to decode the complex interplay between genetic variations and regulatory mechanisms. Such pursuits hold promise for expanding our knowledge of *Leishmania* biology and, by extension, our capacity to manage and mitigate the impact of therapeutic interventions.

## Supporting information

S1 Fig Drug dose-response curves of *Leishmania orientalis* strain PCM2 promastigote under amphotericin B at a concentration of 0.01 to 100 μm, as measured by the colourimetric methods using Alamar Blue as an indicator. (TIF)

S2 Fig Estimation of genome ploidy based on allele frequency ratios in *Leishmania orientalis* strain PCM2 control (A) and treated with 0.3 μm amphotericin B (B). The frequency ratio of heterozygous positions is represented on the X axis, whereas the Y axis indicates the number of heterozygous positions with a given allele frequency ratio. The greater peak in 0.5 indicates that most heterozygous positions support a diploid genome overall.
(TIF)

S3 Fig Principal Component Analysis (PCA) of RNA-seq data from AmB-treated and control samples. The PCA plot shows the distribution of biological replicate samples under two conditions: AmB-treated (in red) and control (in blue). The x-axis represents the first principal component (PC1), and the y-axis represents the second principal component (PC2). Each label corresponds to a biological replicate (LS1, LS2, and LS3) for both conditions.
(TIF)

S1 Table Mean Read Depth by chromosome and gene for control and AmB treatment conditions. (XLSX)
