# Supplementary figures and images for: Genome alteration of Leishmania orientalis under Amphotericin B inhibiting conditions
Source: PLoS Negl Trop Dis. 2024 Dec 17;18(12):e0012716. doi: 10.1371/journal.pntd.0012716 (PMC11687891; doi:10.1371/journal.pntd.0012716)

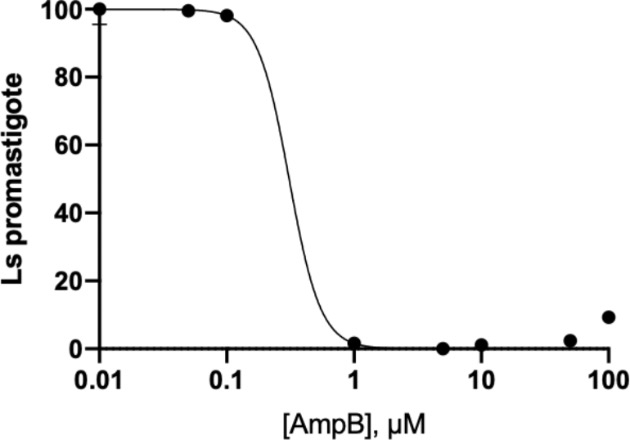

Supplement: S1 Fig — (TIF) [file pntd.0012716.s001.tif]

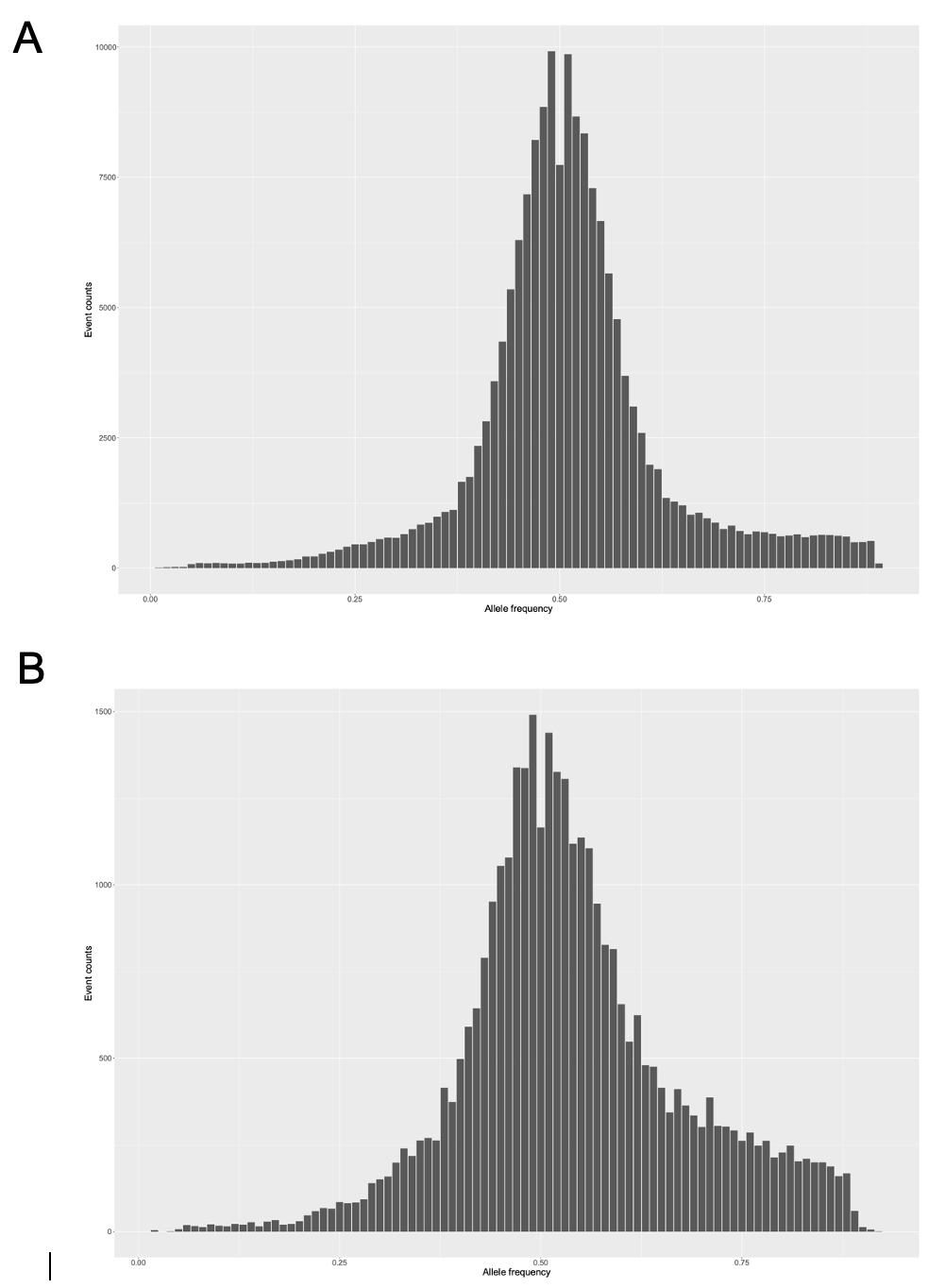

Supplement: S2 Fig — Estimation of genome ploidy based on allele frequency ratios in Leishmania orientalis strain PCM2 control (A) and treated with 0.3 μm amphotericin B (B). The frequency ratio of heterozygous positions is represented on the X axis, whereas the Y axis indicates the number of heterozygous positions with a given allele frequency ratio. The greater peak in 0.5 indicates that most heterozygous positions support a diploid genome overall. (TIF) [file pntd.0012716.s002.tif]

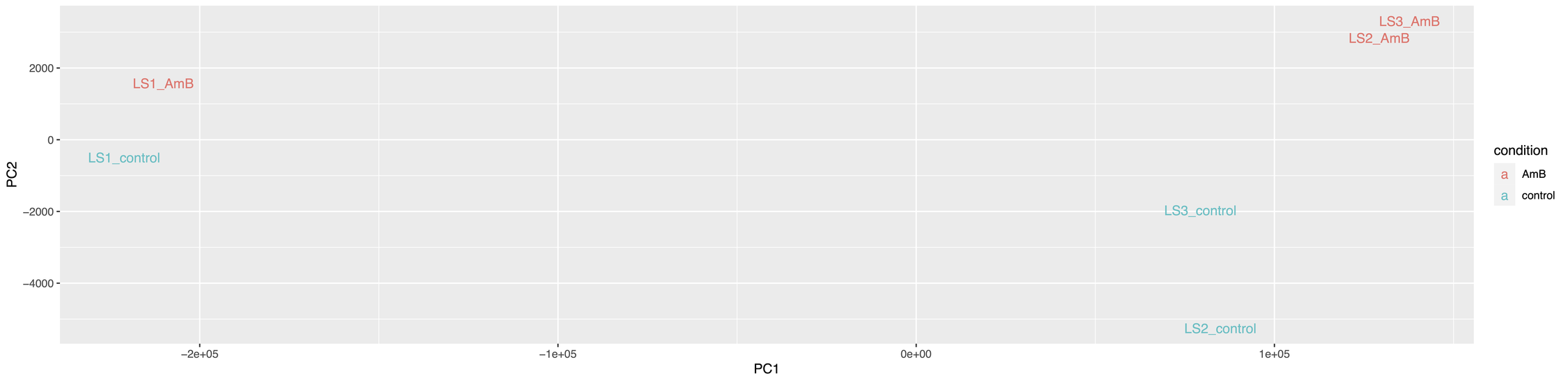

Supplement: S3 Fig — The PCA plot shows the distribution of biological replicate samples under two conditions: AmB-treated (in red) and control (in blue). The x-axis represents the first principal component (PC1), and the y-axis represents the second principal component (PC2). Each label corresponds to a biological replicate (LS1, LS2, and LS3) for both conditions. (TIF) [file pntd.0012716.s003.tif]
